# Type II Peter’s anomaly with histopathological proof: a case report

**DOI:** 10.1186/s12886-017-0502-7

**Published:** 2017-06-29

**Authors:** Rui-Qi Chang, Yu Du, Xiang-Jia Zhu, Yi Lu

**Affiliations:** 1 Department of Ophthalmology, Eye and Ear, Nose, and Throat Hospital, Fudan University, 83 Fenyang Road, Shanghai, 200031 China; 2 Eye Institute, Eye and Ear, Nose, and Throat Hospital, Fudan University, 83 Fenyang Road, Shanghai, 200031 China; 3 Key Laboratory of Myopia, Ministry of Health, 83 Fenyang Road, Shanghai, 200031 China; 4 Key Laboratory of Visual Impairment and Restoration of Shanghai, Fudan University, 83 Fenyang Road, Shanghai, 200031 China

**Keywords:** Peter’s anomaly, Congenital cataract, Corneal leukoma, Amblyopia, Histopathology, Corneolenticular adhesion

## Abstract

**Background:**

Peter’s anomaly is a rare congenital anterior segment dysgenesis with poor visual results. This case report describes a case of bilateral Type II Peter’s anomaly with notable clinical and histopathological features.

**Case presentation:**

A 7-year-old boy was admitted to our center with complaints of bilateral central opacification, photophobia and severe reduced vision since birth. He underwent phacoemulsification, intraocular lens (IOL) implantation and anterior vitrectomy on the right eye in another medical institution two years ago. Slit lamp examination revealed bilateral central corneal opacity, few strands of peripheral iris, irregular pupils and cloudy lens with central adhesion to posterior corneal surface in the left eye. Additionally, a history of premature birth and mental retardation was also noted. The patient was diagnosed with Peter’s anomaly in the left eye, pseudophakia in the right eye and bilateral amblyopia. Similar surgery to the right one was performed on the left eye. A vesicle-like structure was found in the anterior chamber intraoperatively, which was composed mainly of immature lens and some corneal stroma as revealed by postoperative histopathological examinations.

**Conclusions:**

The exact mechanism of Peter’s anomaly is not completely understood, however, the notable histopathological features of tissue obtained from the present case may provide evidence to the hypothesis of developmental anomalies.

## Background

Peter’s anomaly [1] is a rare congenital ocular anomaly mostly autosomal recessive inherited. Approximately 80% of the cases are bilateral. In 1906, Peter first reported a series of anterior segment dysgenesis which consisted of central corneal leukoma and absence of posterior corneal stroma or Descemet membrane, with varying degrees of iris or lenticular adhesions to the posterior corneal surface [2]. The most common concomitant ocular abnormalities were glaucoma (20%), microphthalmos (18%), coloboma (6%) [3], and microcornea [4].

Herein, we report a case of bilateral type II Peter’s anomaly. In particular, we obtained a vesicle-like structure histopathologically confirmed to be a complex of predominantly immature lens and corneal stroma partially.

## Case presentation

A 7-year-old boy was admitted to our center with a main complaint of bilateral central opacification, photophobia and severe reduced vision since birth. His medical history included childhood mental retardation and phacoemulsification, IOL implantation and anterior vitrectomy on the right eye in 2014 in another medical institution. At examination, visual acuity was counting fingers (CF) in the right eye and light perception (LP) in the left eye. The intraocular pressure was 21 mmHg in the right eye and 23 mmHg in the left eye. Slit lamp examination revealed bilateral nystagmus with no conjunctival congestion, diffuse corneal opacity of the right eye and central corneal opacity of the left eye, few strands of peripheral iris and irregular pupils in both eyes, normal IOL position in the right eye and cloudy lens with central adhesion to the posterior corneal surface in the left eye. B-scan ultrasound failed to show other abnormalities in either of the eyes. The patient was not cooperative in further ophthalmic examinations. General physical examination was noncontributory. The boy was the first child delivered normally by the mother at preterm. He was born a gestational period of 35 weeks with a birth weight of 2.51 kg. The Apgar score was 9, and he did not go through oxygen chamber. The mother denied infection or medication history in pregnancy. Moreover, no familial predisposition to any disease was identified.

**Fig. 1:**
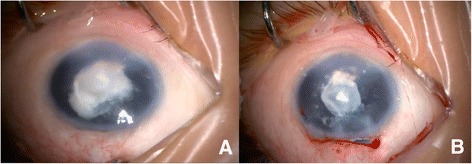
Intraoperative findings of the left eye. **a** The central corneal opacity with the cloudy, irregular lens attached to the central aspect of the posterior cornea. **b** The white, translucent, vesicle-like structure with the central milky opaque patch which closely stuck to the posterior cornea appeared after the phacoemulsification of partial peripheral cortex

**Fig. 2:**
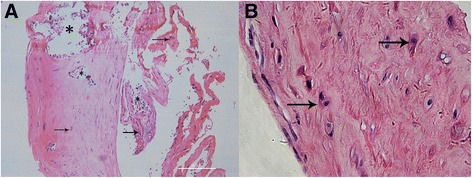
Hematoxylin-Eosin staining of the vesicle-like structure separated from lens. **a** The fibrous tissue with fibroblasts, proliferated collagenous fiber, scattered epithelial-like (*arrow*), broken and degenerated lens cortex accompanied by calcification (*) in the central focal region, and absent Descemet membrane and lens capsule. (Hematoxylin-Eosin stain, original magnification ×10). **b** The cubical epithelial-like cells (*arrow*). (Hematoxylin-Eosin stain, original magnification ×40)

**Fig. 3:**
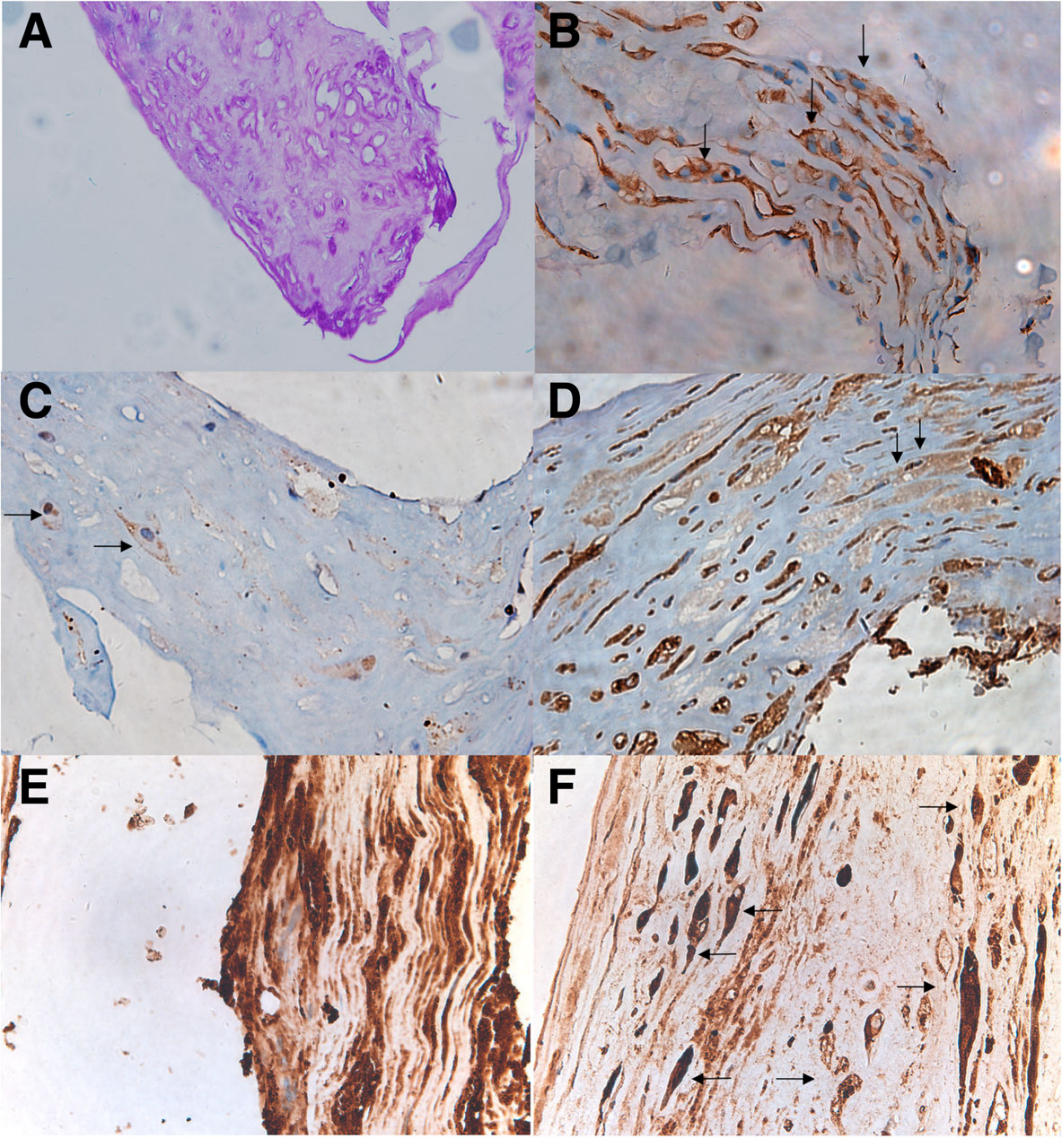
Histological photographs of the vesicle-like structure from lens. **a** Descemet membrane and lens capsule was absent in this structure. (Periodic Acid-Schiff stain, original magnification ×5). **b** The scattered, distributed, epithelial-like cells (*arrow*) stained positively for cytokeratins. (Immunohistochemical stain for cytokeratins, original magnification ×40). **c** The scattered, distributed, epithelial-like cells (*arrow*) stained positively for S-100 protein. (Immunohistochemical stain for S-100 protein, original magnification ×40). **d** The scattered, distributed, epithelial-like cells (*arrow*) stained positively for vimentin. (Immunohistochemical stain for vimentin, original magnification ×40). **e** The lens fibers with intense positive immunoreactivity for alpha-crystallin. (Immunohistochemical stain for alpha- crystallin, original magnification ×40). **f** The scattered, distributed, epithelial-like cells (*arrow*) stained positively for alpha-crystallin. (Immunohistochemical stain for alpha-crystallin, original magnification ×40)

The patient was diagnosed with type II Peter’s anomaly in the left eye, pseudophakia in the right eye and bilateral amblyopia. He was then treated by operations including phacoemulsification, IOL implantation and anterior vitrectomy on the left eye under general anesthesia. During surgery, a cloudy, irregular lens, which adhered to the central posterior corneal surface, was seen. After the phacoemulsification of partial peripheral cortex, the white, translucent, vesicle-like structure with the central milky opaque patch which closely stuck to the posterior cornea appeared (Fig. 1). Postoperative hematoxylin-eosin (HE) staining revealed fibrous tissue with proliferated collagenous fiber and fibroblasts, scattered epithelial-like cells which were spindle or cuboidal, broken and degenerated lens cortex in the central focal region, absent Descemet membrane and lens capsule (Fig. 2). Periodic Acid-Schiff staining confirmed the absence of Descemet membrane and lens capsule (Fig. 3a). In order to identify the cell types and tissue origin of epithelial-like cells, the immunohistochemical staining were then performed, which revealed cytokeratins (CK), S-100, vimentin (VIM) and alpha-crystallin positive cells (Fig. 3b–f). Based on the micromorphology of HE and immunohistochemical staining, the epithelial-like cells were more likely to be lens epithelial cells [5].

On the first day postoperatively, the visual acuity was hand movement (HM) in the left eye, and the intraocular pressure was normal. Slit lamp examination revealed mild conjunctival congestion, central corneal opacity, normal depth of anterior chamber, few strands of peripheral iris and normal IOL position in the left eye. The right eye was in the same condition as on admission.

## Discussion

Peter’s anomaly was subdivided into 2 types [6]. Type I is defined as the presence of the central corneal opacity with iridocorneal adhesions, in which the lens may or may not be cataractous, whereas type II is defined as the presence of cataract or corneolenticular adhesion. In this case, the patient had type II for the presence of cataract and extensive corneolenticular adhesion.

We report positive immunoreactivity for cytokeratin, S-100 protein, the intermediate filament vimentin, and alpha-crystallin. It has been demonstrated that human lenticular intermediate filaments consist exclusively of vimentin, and the protein is predominantly cytoplasmic in the normal lens epithelial cells [7, 8]. Korsakova et al. found a positive immunohistochemical reaction to S-100 in the cortical layers of the lens with cortical cataract, though negative reaction in intact lenses [9]. Also, it was reported that cytokeratin was present in the developing human lens epithelium during early fetal life, and it gradually decreased with age [10, 11]. Alpha-crystallin is one of the major lens-specific protein, and it was found in epithelial cells and the fiber cells [12, 13]. In this case, Immunohistochemical staining of these epithelial-like cells revealed the presence of cytokeratin, S-100 protein, the intermediate filament vimentin, and alpha-crystallin which were considered to be derived from lens epithelial cells. Some fibrous tissue, negative of alpha-crystallin staining and existing in the outer layer of the vesicle-like structure, were recognized as partial corneal stroma.

Although the exact pathogenesis of Peter’s anomaly is not completely understood, the hypothesis of developmental anomalies was already proposed. From embryologic perspective, the clinical features in Peter’s anomaly were thought to derive from the abnormal development of ocular anterior segment, including mesodermal layer which develops into corneal endothelium, corneal and iris stroma, perhaps accompanied by dysplastic lens vesicle as well [14]. It is now known that no mesoderm is involved. In fact, the neural crest cells form the corneal endothelium and keratocytes, iris stroma cells and melanocytes, trabecular meshwork, and juxtacanalicular tissue, following the migration between the surface ectoderm and the periphery of the optic cup [15]. In this case, the white, translucent, vesicle-like structure separated from the irregular lens was confirmed by histopathology as a complex of immature lens for the deformed lens structure with the absence of lens capsule, and the incompletely differentiated lens fiber. This obvious dysgenesis of lens may support the embryologic hypothesis. In addition, the extensive corneolenticular adhesions reflected the abnormal separation between lens and cornea during the embryonic development [16], which is another possible proof for the hypothesis. The secondary endothelial degeneration caused by late anterior displacement of the lens may also be responsible for the occurrence of Peter’s anomaly [13, 14]. The histopathological evaluation which indicated an extensive corneolenticular adhesion with retrocorneal fibrous tissue fill the central defect of corneal stroma may be an evidence for it [17]. Besides, the potential association between Peter’s anomaly and the history of premature birth or childhood mental retardation was worth noting. Kim et al. reported a premature female infant suffered from the spontaneous corneal perforation in an eye with Peters’ anomaly. She was born a gestational period of 33 weeks with a birth weight of 1.41 kg [18]. Also, Myles et al. reported a female infant with Peter’s anomaly who is born at a gestational period of 35 weeks [19]. Normally, the lens vesicle separates from the overlying surface ectoderm by the 6th week of gestation in the human, followed by the primitive endothelium formation for the cornea. By the 20th week of gestation, the anterior chamber is well defined [15, 20], which is much earlier than preterm birth at 35 weeks of gestation. It is possible that both premature birth and Peter’s anomaly are due to some abnormality of fetus.

As for Peter’s anomaly, in principal, once diagnosed, the operation should be done as early as possible to avoid form deprivation [21]. For this case, the treatment for congenital cataract was our focus. Phacoemulsification is the main strategy for the treatment of cataract [22, 23]. Whether the IOL implantation will be performed depends on the age and ocular condition of patient [18, 19]. By measuring the size, location, and density of the opacity, penetrating keratoplasty may be considered, especially when the opacity obstructs the optical pathway [24]. Meanwhile, postoperative optical correction and occlusion therapy are necessary for better visual recovery. In this case, the patient had bilateral type II Peter’s anomaly, and went through the phacoemulsification, IOL implantation and anterior vitrectomy on the right eye in 2014. The preoperative vision was LP of the right eye. On the first day postoperatively, the visual acuity was HM. And the visual acuity of the right eye changed to CF at final follow-up. The prognosis and recovery of his right eye could be an helpful guidance for his treatment of the left eye.

## Conclusions

Peter’s anomaly has been reported for several times, but to the best of our knowledge, this is the first reported case with such histological evidence. Here we obtained a vesicle-like structure which is confirmed by histopathology as a complex of immature lens mainly and corneal stroma partially. The histological findings may contribute to the pathogenesis of Peter’s anomaly.

## Abbreviations

CF: Counting fingers
CK: Cytokeratins
HE: Hematoxylin-Eosin
HM: Hand movement
IOL: Intraocular lens
LP: Light perception
VIM: Vimentin
